# Venous Thromboembolism in Severe Renal Insufficiency: 20 Years of Contemporary Management and Clinical Outcomes

**DOI:** 10.1016/j.jacadv.2026.103268

**Published:** 2026-09-23

**Authors:** Patricia Sigüenza, Juan José López-Núñez, Luis Hernández-Blasco, Ma Luisa Peris, Covadonga Gómez-Cuervo, José Ferreiro-Celeiro, Juan Esteban Gómez-Mesa, David Jiménez, Behnood Bikdeli, Manuel Monreal, the RIETE Investigators

**Affiliations:** a Department of Internal Medicine, Hospital Germans Trias i Pujol, Badalona, Barcelona, Spain; b Department of Medicine, Universitat Autònoma de Barcelona, Barcelona, Spain; c Institut de Recerca Germans Trias i Pujol, Barcelona, Spain; d CIBER Enfermedades Respiratorias (CIBERES), Madrid, Spain; e Pneumology Department of Clinical Medicine, Miguel Hernandez University, ISABIAL, Dr. Balmis University General Hospital, Alicante, Spain; f Department of Internal Medicine, Consorcio Hospitalario Provincial de Castellón, Castellón, Spain; g Universidad Cardenal Herrera-CEU, CEU Universities, Alfara del Patriarca, Spain; h Department of Internal Medicine, Hospital Universitario 12 de Octubre, Madrid, Spain; i Department of Internal Medicine, Hospital Universitario San Agustín, Avilés, Asturias, Spain; j Department of Internal Medicine, Hospital Universitario Fundación Valle del Lili, Cali, Colombia; k Respiratory Department, Hospital Ramón y Cajal and Instituto Ramón y Cajal de Investigación Sanitaria IRYCIS, Madrid, Spain; l Medicine Department, Universidad de Alcalá, Madrid, Spain; m Division of Cardiovascular Medicine, Brigham and Women’s Hospital, Harvard Medical School, Boston, Massachusetts, USA; n Thrombosis Research Group, Brigham and Women’s Hospital, Harvard Medical School, Boston, Massachusetts, USA; o Center for Outcomes Research and Evaluation (CORE), Yale New Haven Hospital, New Haven, Connecticut, USA; p Chair for the Study of Thromboembolic Disease, Faculty of Health Sciences, UCAM-Universidad Católica San Antonio de Murcia, Murcia, Spain

**Keywords:** anticoagulation, major bleeding, real-world practice, severe renal insufficiency, treatment patterns, venous thromboembolism

## Abstract

**Background:**

Patients with severe renal insufficiency have been systematically excluded from randomized trials of anticoagulation for venous thromboembolism (VTE), leaving uncertainty regarding their management in clinical practice.

**Objectives:**

The purpose of this study was to describe the clinical profile, temporal trends in management, and phase-specific outcomes of patients with acute VTE and severe renal insufficiency.

**Methods:**

We analyzed 5,763 patients with acute VTE and creatinine clearance < 30 mL/min enrolled in Registro Informatizado de Enfermedad TromboEmbólica (2005-2024). Baseline characteristics, anticoagulant strategies, and 90-day outcomes were evaluated across 4 calendar periods and during the early (days 0-10) and late (days 11-90) treatment phases.

**Results:**

Mean age was 81 years; 69% were women, 56% presented with pulmonary embolism (PE), 16% had creatinine clearance <15 mL/min, and 3.6% were receiving hemodialysis. Over time, severe renal dysfunction and hemodialysis became more frequent. Initial treatment remained predominantly low-molecular-weight heparin, whereas vitamin K antagonist use declined and direct oral anticoagulant use increased. At 90 days, recurrent VTE occurred in 1.3% of patients, major bleeding in 5.1%, and 20% died. During the first 10 days, fatal PE exceeded fatal bleeding (2.0% vs 0.3%), whereas during days 11 to 90 fatal bleeding exceeded fatal PE (0.7% vs 0.3%).

**Conclusions:**

Patients with VTE and severe renal insufficiency are elderly, medically complex, and experience high short-term mortality. The relative balance between fatal PE and fatal bleeding changes over time, with fatal PE predominating early and fatal bleeding thereafter, while real-world management frequently differs from product labels and guideline recommendations.

Venous thromboembolism (VTE) is a major cause of cardiovascular morbidity and mortality worldwide. Its management becomes particularly challenging in patients with severe renal insufficiency, who represent an increasingly frequent and clinically vulnerable population.^1^, ^2^, ^3^ Impaired renal function is associated with advanced age, multiple comorbidities, altered pharmacokinetics of anticoagulant drugs, and an increased risk of both recurrent VTE and major bleeding.^3^, ^4^, ^5^, ^6^, ^7^, ^8^ Consequently, therapeutic decisions in these patients require balancing the prevention of fatal pulmonary embolism (PE) against the risk of serious hemorrhagic complications.

Despite their high clinical risk, patients with severe renal insufficiency have been systematically excluded from randomized trials evaluating anticoagulant therapy for acute VTE.^9^, ^10^, ^11^, ^12^ As a result, current recommendations rely largely on pharmacokinetic studies, observational evidence, and expert opinion rather than robust comparative clinical data. Furthermore, product labels, regulatory recommendations, and professional society guidelines are not fully concordant regarding the use and dosing of anticoagulants in this setting.^13^, ^14^, ^15^ For example, recommendations differ with respect to dose-adjusted low-molecular-weight heparin (LMWH), vitamin K antagonists (VKAs), and direct oral anticoagulants (DOACs), particularly in patients with creatinine clearance (CrCl) <15 mL/min or those receiving chronic hemodialysis. This uncertainty inevitably influences routine clinical practice.

During the past 2 decades, anticoagulant therapy for VTE has evolved considerably. The introduction of DOACs has transformed the management of most patients with VTE, yet their role in severe renal insufficiency remains controversial.^13^, ^14^, ^15^ Consequently, little is known about how physicians actually manage these patients in daily practice, how treatment strategies have changed over time, or whether prescribing patterns follow current product labeling and guideline recommendations. Likewise, the temporal evolution of thrombotic and bleeding complications during anticoagulant therapy in this population has not been well characterized.

Using data from the international RIETE (Registro Informatizado de Enfermedad TromboEmbólica) registry, we sought to describe the clinical characteristics, management strategies, and 90-day outcomes of patients with acute VTE and severe renal insufficiency enrolled over a 20-year period. Specifically, we aimed to evaluate temporal trends in patient characteristics, anticoagulant selection and dosing, the concordance of real-world management with product-label and guideline recommendations, and the timing of recurrent VTE, major bleeding, and mortality during anticoagulant therapy.

## Methods

### Study design and patients

The RIETE is an ongoing, international, multicenter, prospective registry of consecutive patients with objectively confirmed acute VTE. Patients are enrolled from both inpatient and outpatient settings and are managed according to the clinical practice of each participating center. The rationale and methodology of RIETE have been previously described.^16^ For the present study, we included consecutive patients enrolled between January 2005 and December 2024 who had acute symptomatic deep vein thrombosis (DVT) and/or PE and severe renal insufficiency, defined as CrCl <30 mL/min at the time of VTE diagnosis. Renal function was estimated using the Cockcroft-Gault equation. Patients participating in blinded therapeutic trials were excluded.

DVT was confirmed by compression ultrasonography or venography, and PE by computed tomography pulmonary angiography, ventilation-perfusion lung scanning, or pulmonary angiography. Patients were followed for at least 90 days or until death. All patients provided informed consent in accordance with local requirements, and the study was approved by the ethics committees of participating centers.

### Data collection

Baseline variables (at the time of diagnosis) included demographics, initial VTE presentation (PE with or without concomitant DVT, isolated lower-limb DVT, or isolated upper-limb DVT), vital signs, comorbidities, VTE risk factors, laboratory parameters, renal function, and concomitant medications. Treatment data included anticoagulant class, dosing regimen, duration of therapy, and transitions between treatments during follow-up.

Severe renal insufficiency was defined as CrCl <30 mL/min. Patients with CrCl <15 mL/min and those receiving chronic hemodialysis were additionally evaluated as clinically relevant subgroups.

### Treatment phases and temporal trends

Clinical outcomes were evaluated according to 2 predefined treatment phases: an early phase (days 0-10) and a late phase (days 11-90) after VTE diagnosis. These intervals were selected to distinguish the acute treatment period, when thrombotic risk is highest, from the subsequent period during which bleeding complications may become more prominent. This approach is also consistent with previous RIETE analyses demonstrating a time-dependent pattern of thrombotic and hemorrhagic complication.^17^^,^^18^

To describe temporal changes in patient characteristics and management, patients were categorized into 4 calendar periods: 2005 to 2009, 2010 to 2014, 2015 to 2019, and 2020 to 2024.

### Concordance with product labels and clinical guidelines

Because recommendations for anticoagulant therapy in severe renal insufficiency differ between product labels and clinical practice guidelines, concordance with each source was evaluated separately.

Label-concordant treatment was defined as the use of an anticoagulant and dosing regimen consistent with the approved product information according to the patient’s renal function. Guideline-concordant treatment was defined according to the recommendations of the clinical practice guidelines applicable to VTE treatment in patients with severe renal insufficiency. Treatment strategies not meeting these criteria were classified as label- or guideline-discordant, respectively.

For LMWH and DOACs, dosing patterns were further described using reference regimens based on commonly used prescribing information for VTE treatment in patients with renal impairment. For LMWH in patients with CrCl 15 to 30 mL/min, reference reduced-dose regimens included enoxaparin 1 mg/kg once daily or 0.5 mg/kg twice daily; dalteparin 200 IU/kg once daily; tinzaparin 175 IU/kg once daily; and bemiparin 85 IU/kg once daily. Doses within ±20% of these reference regimens were categorized as “reference dose,” and all other regimens were categorized as “other doses.”

For DOACs, reference regimens in patients with CrCl 15 to 30 mL/min included rivaroxaban 15 mg twice daily and apixaban 10 mg twice daily during the early phase, and rivaroxaban 15 mg once daily, apixaban 5 mg twice daily, or edoxaban 30 mg once daily during the late phase. Doses not corresponding to these regimens were categorized as “other doses.” Use of DOACs in patients with CrCl below 15 mL/min or receiving hemodialysis was described separately.

### Outcomes

The primary outcomes were all-cause mortality and major bleeding within 90 days after VTE diagnosis. Secondary outcomes included recurrent VTE, fatal PE, and fatal bleeding. Outcomes were evaluated overall, across calendar periods, and separately during days 0 to 10 and days 11 to 90. Major bleeding was defined as overt bleeding requiring transfusion of 2 or more units of blood, bleeding at a critical site (retroperitoneal, spinal, intraocular, or intracranial), or fatal bleeding. This definition has been used prospectively in RIETE since the registry was initiated in 2001, before publication and widespread adoption of the International Society of Thrombosis and Haemostasis (ISTH) major bleeding definition. Because all components required to retrospectively apply ISTH criteria were not systematically collected throughout the study period, bleeding events could not be reliably reclassified according to ISTH criteria.

Recurrent VTE was defined as any new symptomatic and objectively confirmed DVT or PE. Fatal PE and fatal bleeding were defined as deaths occurring within 10 days of the respective event in the absence of an alternative cause. Other causes of death were classified according to the information reported by the participating investigators.

### Statistical analysis

Continuous variables were expressed as mean ± SD or median (IQR), as appropriate, and categorical variables as frequencies and percentages. Baseline characteristics, treatment patterns, and clinical outcomes were compared across the 4 calendar periods to evaluate temporal trends.

Clinical outcomes were calculated for the overall 90-day follow-up and separately for the early (days 0-10) and late (days 11-90) phases. Outcomes were also evaluated according to VTE presentation, severity of renal dysfunction, chronic hemodialysis, active cancer, anticoagulant strategy, and concordance of prescribed treatment with product-label and guideline recommendations.

Comparisons of categorical variables were performed using the chi-square test or Fisher exact test, as appropriate, and continuous variables using Student’s *t*-test, analysis of variance, or the corresponding nonparametric tests. Temporal trends in categorical variables were assessed using the Cochran-Armitage test for trend, whereas trends in continuous variables were assessed using linear regression or analysis of variance, as appropriate. Because treatment allocation and dosing were not randomized but determined by treating physicians according to clinical characteristics, comparisons between anticoagulant strategies were considered descriptive and were not intended to estimate comparative treatment effects. Therefore, no formal comparative effectiveness analyses were performed. All statistical analyses were performed using SPSS version 30 (IBM Corp). All tests were 2-sided, and *P* values <0.05 were considered statistically significant.

## Results

### Study population and temporal trends

Between 2005 and 2024, 5,763 patients with acute VTE and severe renal insufficiency were enrolled in the RIETE registry. The mean age was 81 years, 69% were women, and 56% presented with PE (Table 1). Overall, 936 patients (16%) had CrCl <15 mL/min and 209 (3.6%) were receiving hemodialysis.

**Table 1:** Baseline Characteristics Over Time of VTE Patients With Severe Renal Insufficiency

	Total (N = 5,763)	2005-2009 (n = 1,437)	2010-2014 (n = 1,499)	2015-2019 (n = 1,559)	2020-2024 (n = 1,268)	*P* Value for Trend
Female	3,953 (69%)	1,011 (70%)	1,040 (69%)	1,062 (68%)	840 (66%)	0.009
Age, y, mean ± SD	81 ± 12	82 ± 10	82 ± 12	82 ± 12	80 ± 15	<0.001
Body weight, kg/m^2^, mean ± SD	64 ± 14	62 ± 13	64 ± 14	64 ± 14	65 ± 14	<0.001
VTE presentation						
Pulmonary embolism	3,205 (56%)	775 (54%)	828 (55%)	811 (52%)	791 (62%)	0.002
sPESI = 0	323 (5.6%)	60 (4.2%)	73 (4.9%)	77 (4.9%)	113 (8.9%)	<0.001
SBP levels <90 mm Hg	271 (4.7%)	69 (4.8%)	74 (4.9%)	70 (4.5%)	58 (4.6%)	0.680
Lower-limb DVT	2,272 (39%)	617 (43%)	619 (41%)	655 (42%)	381 (30%)	<0.001
Upper-limb DVT	286 (5.0%)	45 (3.1%)	52 (3.5%)	93 (6.0%)	96 (7.6%)	<0.001
Catheter-associated	144 (2.5%)	31 (2.2%)	26 (1.7%)	47 (3.0%)	40 (3.2%)	0.003
Risk factors for VTE						
Active cancer	949 (16%)	253 (18%)	285 (19%)	245 (16%)	166 (13%)	<0.001
Recent surgery	396 (6.9%)	87 (6.1%)	102 (6.8%)	121 (7.8%)	86 (6.8%)	0.163
Immobility for ≥4 days	1,948 (34%)	549 (38%)	514 (34%)	496 (32%)	389 (31%)	<0.001
Hormonal therapy	133 (2.4%)	17 (1.2%)	51 (3.5%)	42 (2.8%)	23 (1.9%)	0.739
Unprovoked	2,769 (48%)	662 (46%)	686 (46%)	757 (49%)	664 (52%)	<0.001
Comorbidities						
Diabetes	1,263 (26%)	154 (24%)	367 (26%)	424 (28%)	318 (26%)	0.142
Hypertension	3,681 (76%)	498 (73%)	1,085 (75%)	1,178 (78%)	920 (75%)	0.086
Prior artery disease	1,343 (28%)	218 (34%)	451 (32%)	383 (26%)	291 (24%)	<0.001
Chronic heart disease	1,025 (18%)	252 (18%)	312 (21%)	251 (16%)	210 (18%)	0.517
Chronic lung disease	709 (12%)	151 (11%)	219 (15%)	190 (12%)	149 (12%)	0.652
Recent major bleeding	198 (3.4%)	47 (3.3%)	46 (3.1%)	64 (4.1%)	41 (3.2%)	0.558
Blood levels						
Anemia	3,388 (59%)	797 (55%)	873 (58%)	954 (61%)	764 (60%)	0.001
Platelet count <100,000/μL	242 (4.2%)	41 (2.9%)	71 (4.7%)	71 (4.6%)	59 (4.7%)	0.034
CrCl levels, mL/min, mean ± SD^a^	21.9 ± 6.3	22.1 ± 6.0	22.1 ± 6.2	22.1 ± 6.3	21.4 ± 6.7	0.002
CrCl levels <15 mL/min	936 (16%)	207 (14%)	235 (16%)	243 (16%)	251 (20%)	<0.001
Hemodialysis	209 (3.6%)	23 (1.6%)	17 (1.1%)	70 (4.5%)	99 (7.8%)	<0.001
Concomitant drugs^b^						
Corticosteroids	573 (15%)	-	199 (14%)	210 (15%)	164 (15%)	0.220
Antiplatelets	428 (11%)	-	173 (14%)	167 (12%)	88 (7.9%)	<0.001
Erythropoietin	206 (5.7%)	-	68 (5.9%)	83 (6.1%)	55 (5.1%)	0.754

CrCl = creatinine clearance; DVT = deep vein thrombosis; RIETE = Registro Informatizado de Enfermedad TromboEmbólica; SBP = systolic blood pressure; sPESI = simplified Pulmonary Embolism Severity Index; VTE = venous thromboembolism.

a CrCl was calculated using Cockcroft-Gault.

b Information on concomitant drugs not recorded in RIETE during the first years.

The clinical profile changed over the 20-year study period (Table 1). The proportion of patients presenting with PE increased from 54% in 2005-2009 to 62% in 2020-2024 (*P* for trend = 0.002), whereas isolated lower-limb DVT declined from 43% to 30% (*P* < 0.001). Upper-limb DVT increased from 3.1% to 7.6% (*P* < 0.001). Patients with CrCl <15 mL/min increased from 14% to 20% (*P* < 0.001), and the proportion receiving hemodialysis increased from 1.6% to 7.8% (*P* < 0.001). Conversely, active cancer decreased from 18% to 13% and recent immobility from 38% to 31%, while unprovoked VTE increased from 46% to 52%. Anemia remained highly prevalent and increased modestly over time (55% to 60%).

### Anticoagulant management and temporal trends

#### Early phase (days 0-10)

Among patients with CrCl 15 to 30 mL/min (n = 4,746), LMWH was the most frequently used treatment category (84%), followed by unfractionated heparin (11%). LMWH use declined modestly over time (87% to 82%), whereas DOAC use, although uncommon overall (1.7%), increased from 0% to 4.7% (*P* < 0.001). Among the 1,017 patients with CrCl <15 mL/min or receiving hemodialysis, LMWH was also the most frequently prescribed therapy (72%), although its use declined from 80% to 65%; unfractionated heparin was used in 21%, and DOAC use increased to 7.6% in the most recent period.

A substantial discordance between guideline- and label-concordant management was observed (Table 2). Among patients with CrCl 15 to 30 mL/min, 97% received a guideline-concordant strategy during the early phase, but only 32% received a label-concordant regimen. Label concordance increased modestly from 28% to 37% over time. In patients with CrCl <15 mL/min or receiving hemodialysis, only 21% received either guideline- or label-concordant therapy.

**Table 2:** Treatment Strategies Over Time

	Total	2005-2009	2010-2014	2015-2019	2020-2024	*P* Value for Trend
Early phase (days 0-10)						
CrCl 15-30 mL/min, N	4,746	1,226	1,255	1,288	977	
Unfractionated heparin	508 (11%)	139 (11%)	149 (12%)	127 (9.9%)	93 (9.5%)	0.023
Low-molecular-weight heparin	4,000 (84%)	1,065 (87%)	1,061 (85%)	1,069 (83%)	805 (82%)	0.005
LMWH, mean doses (IU/kg/d)	162 ± 53	175 ± 47	164 ± 53	155 ± 53	153 ± 58	<0.001
Direct oral anticoagulants	79 (1.7%)	0	2 (0.2%)	31 (2.4%)	46 (4.7%)	<0.001
No anticoagulants	24 (0.5%)	2 (0.2%)	2 (0.2%)	13 (1.0%)	7 (0.7%)	0.004
Guideline-concordant	4,587 (97%)	1,204 (98%)	1,212 (97%)	1,227 (95%)	944 (97%)	0.005
Label-concordant	1,515 (32%)	347 (28%)	365 (29%)	442 (34%)	361 (37%)	<0.001
CrCl <15 or hemodialysis	1,017	211	244	271	291	
Unfractionated heparin	210 (21%)	37 (18%)	48 (20%)	59 (22%)	66 (23%)	0.061
Low-molecular-weight heparin	733 (72%)	168 (80%)	187 (77%)	190 (70%)	188 (65%)	<0.001
LMWH, mean doses (IU/kg/d)	129 ± 62	156 ± 59	137 ± 59	117 ± 59	111 ± 62	<0.001
Direct oral anticoagulants,	24 (2.4%)	0	1 (0.4%)	1 (0.37%)	22 (7.6%)	<0.001
No anticoagulants	12 (1.2%)	1 (0.5%)	1 (0.4%)	6 (2.2%)	4 (1.4%)	0.220
Guideline-concordant	210 (21%)	37 (18%)	48 (20%)	59 (22%)	66 (23%)	0.061
Label-concordant	210 (21%)	37 (18%)	48 (20%)	59 (22%)	66 (23%)	0.061
Late phase (day 11-90)						
CrCl 15-30 mL/min	4,410	1,116	1,173	1,209	912	
Vitamin K antagonists	2,085 (47%)	658 (59%)	664 (57%)	529 (44%)	234 (26%)	<0.001
Low-molecular-weight heparin	1,500 (34%)	390 (35%)	411 (35%)	419 (35%)	280 (31%)	0.135
LMWH, mean doses (IU/kg/d)	143 ± 50	150 ± 51	144 ± 49	143 ± 47	136 ± 51	0.001
Direct oral anticoagulants,	503 (11%)	0	16 (1.4%)	157 (13%)	330 (36%)	<0.001
No anticoagulants	135 (3.1%)	22 (2.0%)	32 (2.7%)	48 (4.0%)	33 (3.6%)	0.011
Guideline-concordant	4,088 (93%)	1,048 (94%)	1,091 (93%)	1,105 (91%)	844 (93%)	0.164
Label-concordant	2,893 (66%)	788 (71%)	792 (68%)	753 (62%)	560 (61%)	<0.001
CrCl <15 or hemodialysis	919	191	220	250	258	
Vitamin K antagonists	375 (41%)	107 (56%)	102 (46%)	89 (36%)	77 (30%)	<0.001
Low-molecular-weight heparin	334 (36%)	65 (34%)	84 (38%)	109 (44%)	76 (29%)	0.226
LMWH, mean doses (IU/kg/d)	127 ± 58	146 ± 70	125 ± 53	126 ± 60	116 ± 48	0.002
Direct oral anticoagulants,	98 (11%)	0	3 (1.4%)	19 (7.6%)	76 (29%)	<0.001
No anticoagulants	44 (4.8%)	5 (2.6%)	11 (5.0%)	18 (7.2%)	10 (3.9%)	0.435
Guideline-concordant	375 (41%)	107 (56%)	102 (46%)	89 (36%)	77 (30%)	<0.001
Label-concordant	375 (41%)	107 (56%)	102 (46%)	89 (36%)	77 (30%)	<0.001

IU = international units; LMWH = low-molecular-weight heparin; other abbreviation as in Table 1.

Detailed dosing patterns further illustrate this discrepancy (Supplemental Table 1). Overall, only 21% of patients received label-concordant LMWH doses, whereas 58% received non-concordant doses. Higher-than-label doses of enoxaparin were particularly frequent (47% of the entire cohort), although their use decreased from 54% in 2005-2009 to 38% in 2020-2024. Specific early-phase DOAC regimens are reported in Supplemental Table 1.

#### Late phase (days 11-90)

Treatment patterns changed substantially during the late phase. Among patients with CrCl 15 to 30 mL/min (n = 4,410), use of VKAs declined from 59% to 26% (*P* < 0.001), whereas DOAC use increased from 0% to 36% (*P* < 0.001); LMWH use remained relatively stable. Similar changes occurred among patients with CrCl below 15 mL/min or receiving hemodialysis (n = 919): VKA use decreased from 56% to 30%, while DOAC use increased from 0% to 29%.

Among patients with CrCl 15 to 30 mL/min, 93% received a guideline-concordant strategy during the late phase, but only 66% received label-concordant therapy, with label concordance decreasing from 71% to 61% over time. In patients with CrCl <15 mL/min or hemodialysis, both guideline- and label-concordant treatment decreased from 56% to 30%. Detailed LMWH and DOAC regimens are provided in Supplemental Table 2. Notably, among DOAC-treated patients, both higher- and lower-than-label doses were used, including higher-dose rivaroxaban and lower-dose apixaban.

### Ninety-day clinical outcomes and trends over time

Overall, 90-day mortality was 20%, major bleeding occurred in 5.1% of patients, and recurrent VTE in 1.3% (Table 3). Mortality declined from 24% in 2005-2009 to 18% in 2020-2024 (*P* < 0.001), accompanied by a reduction in fatal PE from 4.4% to 2.0% (*P* < 0.001). In contrast, major bleeding remained essentially unchanged (5.4% vs 5.3%), as did fatal bleeding. Recurrent PE decreased over time, whereas overall recurrent VTE showed no significant temporal trend.

**Table 3:** Ninety-Day Clinical Outcomes Overall and by Treatment Phase

	All Patients	2005-2009	2010-2014	2015-2019	2020-2024	*P* Value for Trend
Overall (days 0-90)						
Patients	5,763	1,437	1,499	1,559	1,268	
Recurrent VTE	74 (1.3%)	25 (1.7%)	19 (1.3%)	14 (0.9%)	16 (1.3%)	0.097
Recurrent PE	45 (0.8%)	18 (1.3%)	11 (0.7%)	9 (0.6%)	7 (0.6%)	0.010
Recurrent DVT	29 (0.5%)	7 (0.5%)	8 (0.5%)	5 (0.3%)	9 (0.7%)	0.532
Major bleeding	293 (5.1%)	78 (5.4%)	73 (4.9%)	75 (4.8%)	67 (5.3%)	0.630
Death	1,188 (20%)	338 (24%)	313 (21%)	310 (20%)	227 (18%)	<0.001
Fatal PE	130 (2.3%)	63 (4.4%)	19 (1.3%)	23 (1.5%)	25 (2.0%)	<0.001
Fatal bleeding	54 (0.9%)	22 (1.5%)	9 (0.6%)	12 (0.8%)	11 (0.9%)	0.172
Early phase (d 0-10)						
Patients	5,763	1,437	1,499	1,559	1,268	
Recurrent VTE	15 (0.3%)	8 (0.6%)	4 (0.3%)	3 (0.2%)	0	0.008
Major bleeding	128 (2.2%)	35 (2.4%)	31 (2.1%)	32 (2.1%)	30 (2.4%)	0.705
Death	434 (7.5%)	130 (9.0%)	106 (7.1%)	100 (6.4%)	98 (7.7%)	0.042
Fatal PE	114 (2.0%)	56 (3.9%)	17 (1.1%)	19 (1.2%)	22 (1.7%)	<0.001
Fatal bleeding	19 (0.3%)	9 (0.6%)	3 (0.2%)	1 (0.1%)	6 (0.5%)	0.319
Late phase (d 11-90)						
Patients	5,329	1,307	1,393	1,459	1,170	
Recurrent VTE	59 (1.1%)	17 (1.3%)	15 (1.1%)	11 (0.8%)	16 (1.4%)	0.619
Major bleeding	165 (3.1%)	43 (3.3%)	42 (3.0%)	43 (2.9%)	37 (3.2%)	0.688
Death	754 (14%)	208 (16%)	207 (15%)	210 (14%)	129 (11%)	0.001
Fatal PE	16 (0.3%)	7 (0.5%)	2 (0.1%)	4 (0.3%)	3 (0.3%)	0.260
Fatal bleeding	35 (0.7%)	13 (1.0%)	6 (0.4%)	11 (0.8%)	5 (0.4%)	0.314

PE = pulmonary embolism; other abbreviations as in Table 1.

### Phase-specific outcomes

During the first 10 days, 15 patients (0.3%) developed recurrent VTE, 128 (2.2%) experienced major bleeding, and 434 (7.5%) died. Fatal PE occurred in 114 patients (2.0%), compared with 19 fatal bleeding events (0.3%). Thus, although fatal PE accounted for only a minority of all early deaths, it was substantially more frequent than fatal bleeding. Early mortality decreased modestly over time (9.0% to 7.7%), while fatal PE decreased from 3.9% to 1.7%. Major bleeding remained stable.

Among the 5,329 patients alive at day 10, 59 (1.1%) developed recurrent VTE, 165 (3.1%) experienced major bleeding, and 754 (14%) died between days 11 and 90. During this phase, fatal bleeding was more frequent than fatal PE (0.7% vs 0.3%). Late mortality declined from 16% to 11% over the study period (*P* = 0.001), whereas recurrent VTE, major bleeding, fatal PE, and fatal bleeding showed no significant temporal trends.

The temporal accumulation of fatal PE, major bleeding, and fatal bleeding according to the severity of renal dysfunction is shown in Figure 1. Among patients with CrCl 15 to 30 mL/min, fatal PE accumulated predominantly during the first 10 days, whereas major bleeding and fatal bleeding continued to accrue throughout follow-up. This pattern was even more apparent among patients with CrCl <15 mL/min or receiving hemodialysis, in whom the 90-day cumulative incidences of major and fatal bleeding reached 7.3% and 2.1%, respectively.

**Figure 1:**
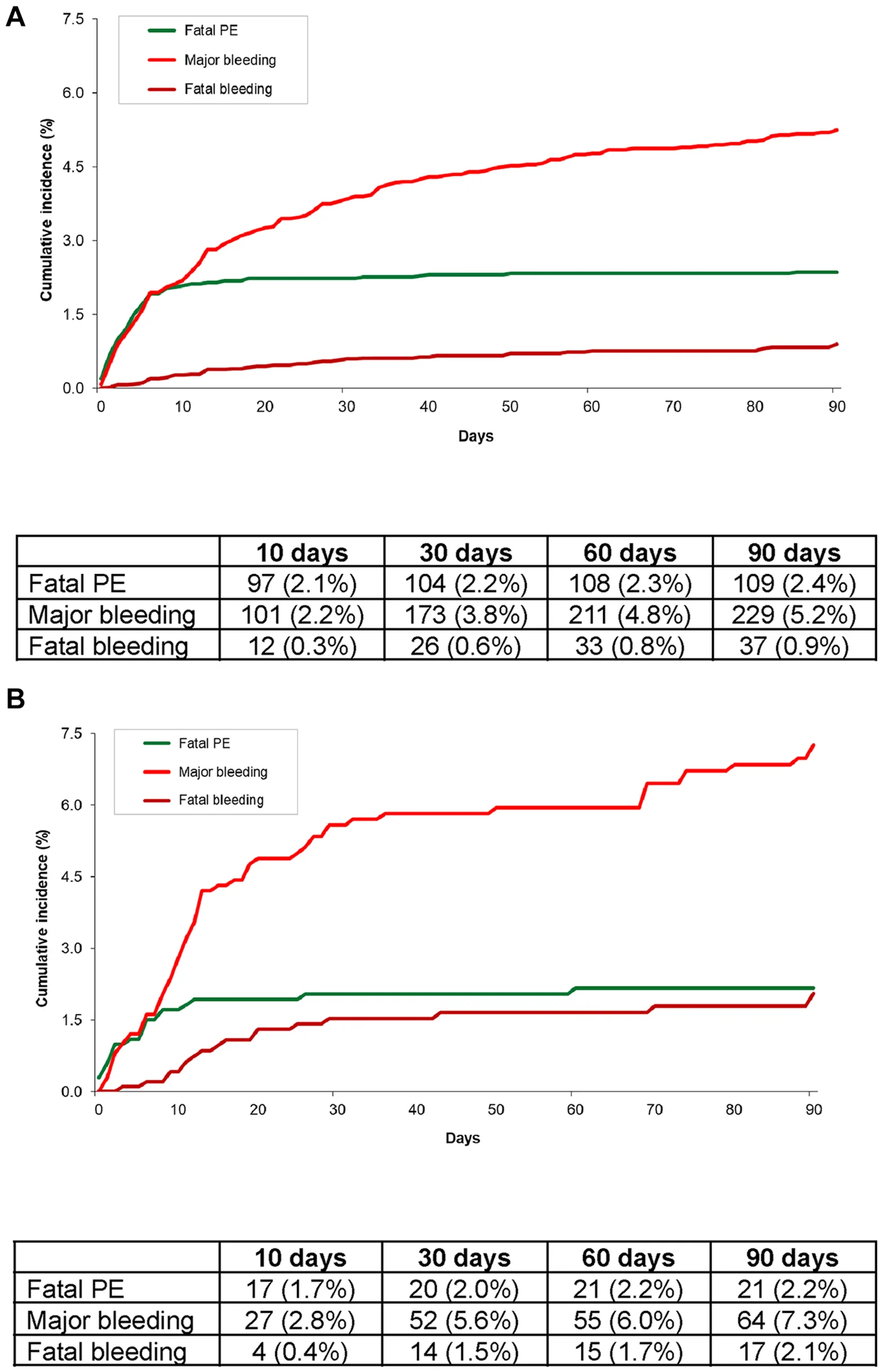
Temporal Distribution of Fatal PE and Bleeding During the First 90 Days After Venous Thromboembolism Diagnosis Cumulative incidence of fatal pulmonary embolism, major bleeding, and fatal bleeding among patients with creatinine clearance 15 to 30 mL/min (A) and those with creatinine clearance <15 mL/min or receiving hemodialysis (B). PE = pulmonary embolism.

### Outcomes according to initial VTE presentation

Outcomes differed according to the initial VTE presentation (Supplemental Table 3). Among patients presenting with PE, early mortality was higher than among those with isolated DVT (8.9% vs 5.8%), largely driven by a higher rate of fatal PE (3.5% vs 0.1%), whereas major bleeding rates were similar. In these patients, early fatal PE declined markedly over time (7.1% to 2.7%, *P* < 0.001), while major bleeding remained stable.

### Outcomes according to anticoagulant strategy and dosing

Unadjusted outcomes according to anticoagulant category, renal function, and dose concordance are shown in Tables 4 and 5. During the early phase, among patients with CrCl 15 to 30 mL/min, major bleeding occurred in 1.2% of those receiving label-concordant LMWH doses and 1.9% receiving other LMWH doses; corresponding mortality rates were 5.7% and 6.5%. Among patients with CrCl <15 mL/min or receiving hemodialysis, major bleeding occurred in 0.4% and 3.5%, respectively.

**Table 4:** Clinical Outcomes According to Anticoagulant Treatment Categories: Early Phase (Days 0-10)

	N	Death	Fatal PE	Major Bleeding
CrCl 15-30 mL/min (N = 4,746)				
Unfractionated heparin	508	48 (9.4%)	14 (2.8%)	24 (4.7%)
LMWH, label-concordant dose	961	55 (5.7%)	15 (1.6%)	12 (1.2%)
LMWH, other doses	3,039	199 (6.5%)	61 (2.0%)	58 (1.9%)
DOACs, label-concordant dose	46	0	0	1 (2.2%)
DOACs, other doses	33	3 (9.1%)	0	0
Other treatments	159	31 (19%)	7 (4.4%)	6 (3.8%)
CrCl <15 mL/min or hemodialysis (N = 1,017)				
Unfractionated heparin	210	24 (11%)	4 (1.9%)	7 (3.3%)
LMWH, label-concordant dose	243	20 (8.2%)	1 (0.4%)	1 (0.4%)
LMWH, other doses	490	41 (8.4%)	9 (1.8%)	17 (3.5%)
DOACs, label-concordant dose	20	0	0	0
DOACs, other doses	4	2 (50%)	0	1 (25%)
Other treatments	50	11 (22%)	3 (6.0%)	1 (2.0%)

Other treatments (early phase): CrCl 15 to 30 mL/min: thrombolytics (n = 30), VKAs (n = 39), fondaparinux (n = 41), other LMWHs (n = 25), no anticoagulation (n = 24).

CrCl <15 mL/min/hemodialysis: thrombolytics (n = 4), VKAs (n = 13), fondaparinux (n = 3), other LMWHs (n = 18), no anticoagulation (n = 12).

VKAs = vitamin K antagonists; DOAC = direct oral anticoagulant; other abbreviations as in Table 1, Table 2, Table 3.

**Table 5:** Clinical Outcomes According to Anticoagulant Treatment Categories: Late Phase (Days 11-90)

	N	Death	Fatal PE	Major Bleeding
CrCl 15-30 mL/min (N = 4,410)				
Vitamin K antagonists	2,085	133 (6.4%)	2 (0.1%)	30 (1.4%)
LMWH, label-concordant dose	529	87 (16%)	1 (0.2%)	17 (3.2%)
LMWH, other doses	971	211 (22%)	4 (0.4%)	48 (5.0%)
DOACs, label-concordant dose	279	16 (5.7%)	0	2 (0.7%)
DOACs, other doses	224	19 (8.5%)	0	4 (1.8%)
Other treatments	322	120 (37%)	5 (1.6%)	27 (9.3%)
CrCl <15 mL/min or hemodialysis (N = 919)				
Vitamin K antagonists	375	40 (11%)	1 (0.3%)	11 (2.9%)
LMWH, label-concordant dose	143	25 (18%)	1 (0.7%)	5 (3.5%)
LMWH, other doses	191	45 (24%)	1 (0.5%)	9 (4.8%)
DOACs, label-concordant dose	27	4 (15%)	0	1 (3.8%)
DOACs, other doses	71	6 (8.5%)	0	1 (1.4%)
Other treatments	112	48 (43%)	1 (0.9%)	10 (10%)

Other treatments (late phase): CrCl 15 to 30 mL/min: UFH (n = 31), fondaparinux (n = 11), other LMWHs (n = 146), no anticoagulation (n = 135).

CrCl <15 mL/min or hemodialysis: UFH (n = 20), fondaparinux (n = 1), other LMWHs (n = 47), no anticoagulation (n = 44).

These comparisons are descriptive and reflect treatment allocation in routine clinical practice.

UFH = unfractionated heparin; other abbreviations as in Table 1, Table 2, Table 3, Table 4.

During the late phase, patients receiving LMWH had higher unadjusted rates of mortality and major bleeding than those receiving VKAs or DOACs. Among patients with CrCl 15 to 30 mL/min, major bleeding occurred in 3.2% with label-concordant LMWH and 5.0% with other LMWH doses, compared with 1.4% with VKAs. Outcomes among DOAC-treated patients were numerically favorable, but patient numbers in several dose categories were small. Similar heterogeneity was observed among patients with CrCl <15 mL/min or receiving hemodialysis. These comparisons are descriptive and should not be interpreted as comparative treatment effects because treatment selection and dosing were determined by clinicians and were strongly influenced by patient characteristics and disease severity.

## Discussion

In this large international cohort of patients with acute VTE and severe renal insufficiency, several findings deserve emphasis. First, these patients were predominantly elderly, frequently women, and highly comorbid, and their clinical profile became increasingly complex over the 20-year study period. Second, anticoagulant management changed substantially, particularly with progressive replacement of VKAs by DOACs, but real-world prescribing frequently differed from product-label recommendations and, to a lesser extent, from clinical practice guidelines. Third, outcomes remained poor: 1 in 5 patients died within 90 days and approximately 1 in 20 experienced major bleeding, whereas recurrent VTE was uncommon. Finally, PE and bleeding showed markedly different temporal patterns. Fatal PE was substantially more frequent than fatal bleeding during the first 10 days, whereas fatal bleeding exceeded fatal PE thereafter. Importantly, both accounted for only a minority of all deaths, highlighting the major contribution of underlying comorbidity and frailty to prognosis (Central Illustration).

**Central Illustration:**
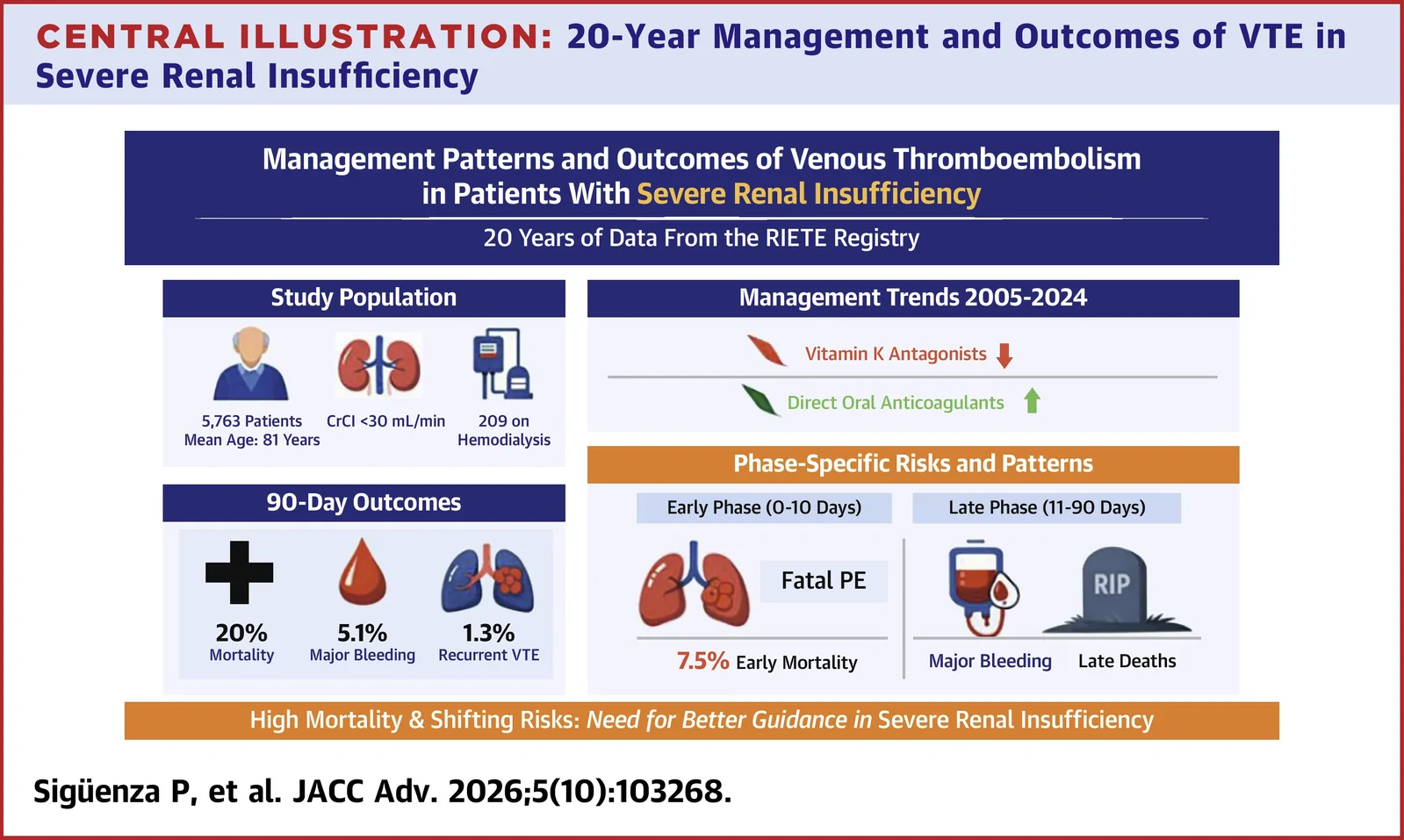
20-Year Management and Outcomes of VTE in Severe Renal Insufficiency Among 5,763 patients with acute venous thromboembolism and severe renal insufficiency (creatinine clearance <30 mL/min), anticoagulant management changed substantially over 20 years, with increasing use of direct oral anticoagulants and declining use of vitamin K antagonists. Despite these changes, important discrepancies persisted between clinical practice, product-labeling, and clinical guidelines. Fatal pulmonary embolism predominated during the first 10 days after diagnosis, whereas fatal bleeding became more frequent thereafter, supporting repeated reassessment of thrombotic and bleeding risks throughout the first 90 days of treatment. CrCl = creatinine clearance; RIETE = Registro Informatizado de Enfermedad TromboEmbólica; VTE = venous thromboembolism; other abbreviation as in Figure 1.

The adverse prognosis observed in our cohort is consistent with previous studies showing that renal dysfunction is a powerful determinant of outcome after VTE. An earlier RIETE analysis showed a graded increase in early mortality, major bleeding, fatal PE, and fatal bleeding as renal function deteriorated; among patients with CrCl <30 mL/min, 15-day mortality reached 16%, compared with 2.3% among those with CrCl >60 mL/min, and major bleeding occurred in 5.4% vs 1.0%, respectively.^17^ More contemporary data from Global Anticoagulant Registry in the FIELD-Venous Thromboembolism (GARFIELD-VTE) similarly showed that patients with moderate-to-severe chronic kidney disease had significantly higher adjusted risks of death, major bleeding, and recurrent VTE than those with mild or no kidney disease.^3^ The 20% 90-day mortality observed in the present study therefore likely reflects both the acute consequences of VTE and the advanced age, severe renal dysfunction, and substantial comorbidity burden of this population.^3^^,^^17^, ^18^, ^19^

An important contribution of the present study is the description of how this population and its management have changed over 2 decades. Although patients remained elderly throughout, the prevalence of CrCl <15 mL/min and hemodialysis increased, suggesting that clinicians are increasingly treating VTE in patients with very advanced kidney disease. At the same time, treatment patterns evolved markedly. LMWH remained the dominant initial therapy, whereas VKA use during long-term treatment declined substantially and DOAC exposure increased, including among patients with CrCl <15 mL/min or receiving hemodialysis. The relatively low overall use of DOACs during the acute phase should be interpreted in the context of the 2005-2024 study period: DOACs were unavailable during the early years and, after their introduction, patients with severe renal insufficiency remained excluded from pivotal VTE trials and their use was restricted or discouraged by product labeling and guidelines.^13^, ^14^, ^15^

Perhaps the most clinically relevant finding is the discordance between recommended and actual anticoagulant management. Among patients with CrCl 15 to 30 mL/min, almost all early treatment strategies were guideline-concordant, yet only approximately one-third were label-concordant. A large proportion of patients receiving LMWH were treated with doses higher than recommended in product labeling, particularly with enoxaparin. Although the frequency of such dosing decreased over time, it remained common even in the most recent period. Conversely, DOACs were increasingly prescribed in patients with very severe renal dysfunction despite limited supporting trial evidence. These findings should not simply be interpreted as inappropriate prescribing. Rather, they illustrate the therapeutic uncertainty faced by clinicians when product labels, regulatory recommendations, professional guidelines, and clinical judgment are not fully aligned.

Several factors may explain why clinicians frequently used higher-than-label LMWH doses. Dose reduction in severe renal insufficiency is intended to limit drug accumulation and bleeding,^4^^,^^5^ but clinicians treating an acute PE may also be concerned that excessive dose reduction could compromise antithrombotic efficacy. This tension is particularly relevant during the earliest phase after VTE diagnosis, when the consequences of undertreatment may be immediate. Indeed, our earlier RIETE analysis reported that, despite increased bleeding risk, fatal PE substantially exceeded fatal bleeding among patients with severe renal insufficiency and supported adequate initial anticoagulation in this setting.^17^ The present study extends those observations by showing how clinicians have actually navigated this uncertainty over 20 years. However, the unadjusted outcome differences according to treatment and dose should not be interpreted as evidence that one strategy is safer or more effective than another, because treatment selection was strongly influenced by clinical status, renal function, perceived thrombotic and bleeding risks, and other factors.

The phase-specific pattern of events provides additional insight. During the first 10 days, fatal PE occurred approximately 6 times more frequently than fatal bleeding (2.0% vs 0.3%). However, fatal PE accounted for only approximately one-quarter of early deaths, and therefore it would be inappropriate to characterize overall early mortality as predominantly PE-related. Rather, PE was the most important anticoagulation-related potentially modifiable cause of early death. After day 10, this relationship reversed: fatal bleeding exceeded fatal PE (0.7% vs 0.3%), while most deaths continued to arise from other causes. Thus, the principal message is not that mortality changes from being “caused by PE” to being “caused by bleeding,” but that the relative balance between the 2 complications most directly affected by anticoagulant therapy changes substantially over time.

This temporal pattern has potential clinical implications. During the acute phase, ensuring adequate anticoagulation may be particularly important, especially in patients presenting with PE. Once the immediate thrombotic hazard declines, the accumulated bleeding risk becomes increasingly relevant, particularly in elderly patients with anemia, severe kidney dysfunction, concomitant antiplatelet therapy, or other comorbidities. These observations support repeated reassessment of anticoagulant choice and dose rather than assuming that the optimal risk-benefit balance remains constant throughout the first 3 months of treatment. They should not, however, be interpreted as evidence supporting systematic dose reduction after day 10; the present observational study was not designed to test such a strategy.

The low incidence of recurrent VTE also deserves comment. Only 1.3% of patients experienced recurrence by 90 days, compared with a 5.1% incidence of major bleeding and 20% mortality. This imbalance emphasizes that, in patients with severe renal insufficiency, the challenge extends beyond preventing recurrent thrombosis. The broader clinical objective is to provide sufficient anticoagulation to prevent potentially fatal PE while limiting hemorrhagic complications in patients with limited physiologic reserve and substantial competing mortality. The higher rates of adverse outcomes reported in patients with chronic kidney disease in GARFIELD-VTE and earlier RIETE studies support the concept that kidney dysfunction identifies a particularly vulnerable VTE phenotype rather than simply modifying the pharmacokinetics of anticoagulants.^3^^,^^17^, ^18^, ^19^

### Strengths

The strengths of the study include its large sample size, international multicenter design, prospective collection of standardized data, and inclusion of consecutive patients treated in routine clinical practice. The 20-year observation period enabled us to characterize major changes in patient phenotype and anticoagulant management, while detailed dose information provided a unique opportunity to compare actual prescribing with product-label and guideline recommendations. The phase-specific analysis also allowed us to distinguish early and later patterns of clinically relevant thrombotic and bleeding events.

### Study Limitations

Several limitations should be acknowledged. First, this was an observational registry, and treatment selection and dosing were determined by treating physicians; therefore, comparisons among anticoagulant strategies are subject to substantial confounding by indication and should not be interpreted causally. Second, renal function was assessed at baseline, and changes in CrCl during follow-up were not systematically incorporated into the analysis. Third, information on anti-Xa monitoring and other measures of anticoagulant intensity was limited. Fourth, RIETE has used its predefined major bleeding definition since the registry began in 2001, before widespread adoption of the ISTH definition; because all components required for retrospective ISTH classification were not systematically collected, we could not reclassify bleeding events according to ISTH criteria. Fifth, causes of death were reported by participating investigators rather than determined by centralized adjudication, and misclassification is possible. Finally, product labeling and clinical guideline recommendations evolved during the 20-year study period and differ across agents and jurisdictions, which should be considered when interpreting concordance analyses.

## Conclusions

Patients with VTE and severe renal insufficiency remain a particularly vulnerable population, characterized by advanced age, increasing prevalence of very severe kidney dysfunction, high short-term mortality, and substantial bleeding risk. Over 20 years, anticoagulant practice changed markedly, yet important discrepancies persisted between product labeling, clinical guidelines, and real-world management. Fatal PE was substantially more frequent than fatal bleeding during the acute phase, whereas this relationship reversed after day 10, although most deaths in both phases were attributable to other causes. These findings highlight the need for repeated, individualized assessment of thrombotic and bleeding risk and, importantly, for prospective studies specifically addressing anticoagulant selection and dosing in patients with severe renal insufficiency.

Perspectives **COMPETENCY IN PATIENT CARE:** Patients with acute VTE and severe renal insufficiency represent a particularly vulnerable population, characterized by advanced age, substantial comorbidity, high short-term mortality, and a considerable risk of major bleeding. The balance between thrombotic and bleeding complications changes over time: fatal PE is substantially more frequent than fatal bleeding during the first 10 days, whereas fatal bleeding exceeds fatal PE thereafter. These findings support repeated reassessment of thrombotic and bleeding risks throughout anticoagulant therapy.

**TRANSLATIONAL OUTLOOK:** Despite major changes in anticoagulant practice over the past 20 years, substantial discordance persists between product-label recommendations, clinical guidelines, and real-world prescribing, reflecting the limited evidence available for patients with severe renal insufficiency. Prospective studies specifically including patients with CrCl <30 mL/min, particularly those with CrCl <15 mL/min or receiving hemodialysis, are needed to define optimal anticoagulant selection and dosing.

## Funding support and author disclosures

Dr Monreal has received unrestricted educational grants for research from ROVI and the Universidad Católica San Antonio de Murcia to sponsor the RIETE registry. All other authors have reported that they have no relationships relevant to the contents of this paper to disclose.
