# Supplementary material for: Venous Thromboembolism in Severe Renal Insufficiency: 20 Years of Contemporary Management and Clinical Outcomes
Source: JACC Adv. 2026 Sep 23;5(10):103268. doi: 10.1016/j.jacadv.2026.103268 (PMC13634757; doi:10.1016/j.jacadv.2026.103268)
Supplement: Supplementary file 1 [file mmc1.docx]

**Supplementary Table 1. Early-Phase Detailed Dosing.**

|  | **Total,**  **N** | **2005-2009** | **2010-2014** | **2015-2019** | **2020-2024** | **p for**  **trend** |
| --- | --- | --- | --- | --- | --- | --- |
| ***Patients, N*** | ***5,763*** | ***1,437*** | ***1,499*** | ***1,559*** | ***1,268*** |  |
| Unfractionated heparin | 718 (12%) | 176 (12%) | 197 (13%) | 186 (12%) | 159 (13%) | 0.791 |
| Low-molecular-weight heparin | 4,733 (82%) | 1,233 (86%) | 1,248 (83%) | 1,259 (81%) | 993 (78%) | <0.001 |
| **LMWH, label-concordant doses**, | **1,204 (21%)** | **248 (17%)** | **271 (18%)** | **370 (24%)** | **315 (25%)** | **<0.001** |
| Enoxaparin 100 IU/kg/day±20% | 737 (13%) | 102 (7.1%) | 175 (12%) | 244 (16%) | 216 (17%) | <0.001 |
| Tinzaparin 175 IU/kg OD±20% | 269 (4.7%) | 76 (5.3%) | 45 (3.0%) | 80 (5.1%) | 68 (5.4%) | 0.375 |
| Bemiparin 85 IU/kg/day±20% | 102 (1.8%) | 17 (1.2%) | 17 (1.1%) | 37 (2.4%) | 31 (2.4%) | <0.001 |
| Dalteparin 200 IU/kg/day±20% | 96 (1.7%) | 53 (3.7%) | 34 (2.3%) | 9 (0.6%) | 0 | <0.001 |
| **LMWH, non-concordant doses**, | **3,354 (58%)** | **875 (61%)** | **950 (63%)** | **874 (56%)** | **655 (52%)** | **<0.001** |
| Enoxaparin, higher doses | 2,693 (47%) | 770 (54%) | 784 (52%) | 662 (42%) | 477 (38%) | <0.001 |
| Enoxaparin, lower doses | 297 (5.2%) | 35 (2.4%) | 75 (5.0%) | 99 (6.4%) | 88 (6.9%) | <0.001 |
| Tinzaparin, higher doses | 39 (0.7%) | 11 (0.8%) | 10 (0.7%) | 14 (0.9%) | 4 (0.3%) | 0.349 |
| Tinzaparin, lower doses | 49 (0.9%) | 9 (0.6%) | 13 (0.9%) | 10 (0.6%) | 17 (1.3%) | 0.077 |
| Bemiparin, higher doses | 223 (3.9%) | 26 (1.8%) | 50 (3.3%) | 82 (5.3%) | 65 (5.1%) | <0.001 |
| Bemiparin, lower doses | 19 (0.3%) | 5 (0.4%) | 5 (0.3%) | 5 (0.3%) | 4 (0.3%) | 0.800 |
| Dalteparin, higher doses | 9 (0.2%) | 6 (0.4%) | 3 (0.2%) | 0 | 0 | 0.009 |
| Dalteparin, lower doses | 25 (0.4%) | 13 (0.9%) | 10 (0.7%) | 2 (0.1%) | 0 | <0.001 |
| Other LMWHs | 175 (3.0%) | 110 (7.7%) | 27 (1.8%) | 15 (1.0%) | 23 (1.8%) | <0.001 |
| Direct oral anticoagulants, | 103 (1.8%) | 0 | 3 (0.2%) | 32 (2.1%) | 68 (5.4%) | <0.001 |
| Rivaroxaban 15 mg BID | 41 (0.7%) | 0 | 3 (0.2%) | 10 (0.6%) | 28 (2.2%) | <0.001 |
| Rivaroxaban, lower doses | 5 (0.1%) | 0 | 0 | 2 (0.1%) | 3 (0.2%) | 0.051 |
| Apixaban, 10 mg BID | 25 (0.4%) | 0 | 0 | 7 (0.5%) | 18 (1.4%) | <0.001 |
| Apixaban lower doses | 30 (0.5%) | 0 | 0 | 13 (0.8%) | 17 (1.3%) | <0.001 |
| Edoxaban 30mg | 1 (0.02%) | 0 | 0 | 0 | 1 (0.1%) | 0.313 |
| Dabigatran 110mg | 1 (0.02%) | 0 | 0 | 0 | 1 (0.1%) | 0.287 |
| Fondaparinux | 44 (0.8%) | 10 (0.7%) | 17 (1.1%) | 14 (0.9%) | 3 (0.2%) | 0.175 |
| Other drugs | 97 (1.7%) | 11 (0.8%) | 25 (1.7%) | 35 (2.2%) | 26 (2.1%) | 0.002 |
| No anticoagulants | 36 (0.6%) | 3 (0.2%) | 3 (0.2%) | 19 (1.2%) | 11 (0.9%) | 0.001 |
|  |  |  |  |  |  |  |

***Abbreviations:*** DVT, deep vein thrombosis; IQR, inter-quartile range; LMWH, low-molecular-weight heparin; IU, international units; BID, twice daily.

**Supplementary Table 2. Late-Phase Detailed Dosing.**

|  | **Total,**  **N** | **2005-2009** | **2010-2014** | **2015-2019** | **2020-2024** | **p for**  **trend** |
| --- | --- | --- | --- | --- | --- | --- |
| ***Patients, N*** | ***5,329*** | ***1,307*** | ***1,393*** | ***1,459*** | ***1,170*** |  |
| Vitamin K antagonists | 2,460 (46%) | 765 (59%) | 766 (55%) | 618 (42%) | 311 (27%) | <0.001 |
| Low-molecular-weight heparin | 1,834 (34%) | 455 (35%) | 495 (36%) | 528 (36%) | 356 (30%) | 0.078 |
| **LMWH, label-concordant doses,** | **672 (13%)** | **150 (11%)** | **155 (11%)** | **197 (14%)** | **170 (15%)** | **0.002** |
| Enoxaparin 100 IU/kg/day±20% | 333 (6.2%) | 65 (5.0%) | 92 (6.6%) | 93 (6.4%) | 83 (7.1%) | 0.041 |
| Tinzaparin 175 IU/kg OD±20% | 243 (4.6%) | 56 (4.3%) | 44 (3.2%) | 78 (5.3%) | 65 (5.6%) | 0.036 |
| Bemiparin 85 IU/kg/day±20% | 75 (1.4%) | 18 (1.4%) | 13 (0.9%) | 22 (1.5%) | 22 (1.9%) | 0.093 |
| Dalteparin 200 IU/kg/day±20% | 21 (0.4%) | 11 (0.8%) | 6 (0.4%) | 4 (0.3%) | 0 | 0.001 |
| **LMWH,non-concordant doses** | **1,091 (20%)** | **255 (20%)** | **328 (24%)** | **328 (22%)** | **180 (15%)** | **0.020** |
| Enoxaparin, higher doses | 707 (13%) | 181 (14%) | 222 (16%) | 205 (14%) | 99 (8.5%) | <0.001 |
| Enoxaparin, lower doses | 142 (2.7%) | 26 (2.0%) | 41 (2.9%) | 47 (3.2%) | 28 (2.4%) | 0.231 |
| Tinzaparin, higher doses | 26 (0.5%) | 5 (0.4%) | 8 (0.6%) | 10 (0.7%) | 3 (0.3%) | 0.947 |
| Tinzaparin, lower doses | 35 (0.7%) | 6 (0.5%) | 8 (0.6%) | 12 (0.8%) | 9 (0.8%) | 0.302 |
| Bemiparin, higher doses | 143 (2.7%) | 17 (1.3%) | 40 (2.9%) | 48 (3.3%) | 38 (3.2%) | <0.001 |
| Bemiparin, lower doses | 21 (0.4%) | 7 (0.5%) | 6 (0.4%) | 5 (0.3%) | 3 (0.3%) | 0.200 |
| Dalteparin, higher doses | 3 (0.1%) | 3 (0.2%) | 0 | 0 | 0 | 0.081 |
| Dalteparin, lower doses | 14 (0.3%) | 10 (0.8%) | 3 (0.2%) | 1 (0.1%) | 0 | 0.001 |
| Other LMWHs | 71 (1.3%) | 50 (3.8%) | 12 (0.9%) | 3 (0.2%) | 6 (0.5%) | <0.001 |
| Direct oral anticoagulants, | 601 (11%) | 0 | 19 (1.4%) | 176 (12%) | 406 (35%) | <0.001 |
| Rivaroxaban 15 mg OD | 55 (1.0%) | 0 | 8 (0.6%) | 18 (1.2%) | 29 (2.5%) | <0.001 |
| Rivaroxaban, higher doses | 137 (2.6%) | 0 | 9 (0.7%) | 54 (3.7%) | 74 (6.3%) | <0.001 |
| Rivaroxaban, lower doses | 2 (0.04%) | 0 | 0 | 1 (0.1%) | 1 (0.1%) | 0.263 |
| Apixaban 5 mg BID | 185 (3.5%) | 0 | 0 | 41 (2.8%) | 144 (12%) | <0.001 |
| Apixaban higher doses | 19 (0.4%) | 0 | 0 | 3 (0.2%) | 16 (1.4%) | <0.001 |
| Apixaban lower doses | 105 (2.0%) | 0 | 1 (0.1%) | 36 (2.5%) | 68 (5.8%) | <0.001 |
| Edoxaban, 30 mg OD | 66 (1.2%) | 0 | 0 | 17 (1.2%) | 49 (4.2%) | <0.001 |
| Edoxaban, 60 mg OD | 18 (0.3%) | 0 | 0 | 4 (0.3%) | 14 (1.2%) | <0.001 |
| Dabigatran | 14 (0.3%) | 0 | 1 (0.1%) | 2 (0.1%) | 11 (0.9%) | <0.001 |
| Fondaparinux | 11 (0.2%) | 2 (0.2%) | 5 (0.4%) | 4 (0.3%) | 0 | 0.401 |
| Other drugs | 244 (4.6%) | 58 (4.4%) | 65 (4.7%) | 67 (4.6%) | 54 (4.6%) | 0.949 |
| No anticoagulants | 179 (3.4%) | 27 (2.1%) | 43 (3.1%) | 66 (4.5%) | 43 (3.7%) | 0.005 |
|  |  |  |  |  |  |  |

***Abbreviations:*** DVT, deep vein thrombosis; IQR, inter-quartile range; LMWH, low-molecular-weight heparin; IU, international units; BID, twice daily; OD, once daily.

**Supplementary Table 3. Clinical Outcomes, by Initial VTE Presentation.**

|  | **All patients** | **2005-2009** | **2010-2014** | **2015-2019** | **2020-2024** | **p for**  **trend** |
| --- | --- | --- | --- | --- | --- | --- |
| **PE patients, Days 0 to 10** | | | | | | |
| ***Patients, N*** | ***3,205*** | ***775*** | ***828*** | ***811*** | ***791*** |  |
| Recurrent VTE | 6 (0.2%) | 3 (0.4%) | 2 (0.2%) | 1 (0.1%) | 0 | 0.063 |
| Major bleeding | 77 (2.4%) | 17 (2.2%) | 18 (2.2%) | 21 (2.6%) | 21 (2.7%) | 0.480 |
| Death | 286 (8.9%) | 89 (11%) | 69 (8.3%) | 58 (7.2%) | 70 (8.8%) | 0.011 |
| Fatal PE | 111 (3.5%) | 55 (7.1%) | 16 (1.9%) | 19 (2.3%) | 21 (2.7%) | <0.001 |
| Fatal bleeding | 10 (0.3%) | 3 (0.4%) | 2 (0.2%) | 1 (0.1%) | 4 (0.5%) | 0.720 |
|  |  |  |  |  |  |  |
| **PE patients, Days 11 to 90** | | | | | | |
| ***Patients, N*** | ***2,919*** | ***686*** | ***759*** | ***753*** | ***721*** |  |
| Recurrent VTE | 25 (0.9%) | 9 (1.3%) | 4 (0.5%) | 6 (0.8%) | 6 (0.8%) | 0.307 |
| Major bleeding | 84 (2.9%) | 14 (2.1%) | 24 (3.2%) | 27 (3.6%) | 19 (2.7%) | 0.495 |
| Death | 394 (13%) | 107 (16%) | 104 (14%) | 100 (13%) | 83 (12%) | 0.025 |
| Fatal PE | 13 (0.4%) | 5 (0.7%) | 1 (0.1%) | 4 (0.5%) | 3 (0.4%) | 0.520 |
| Fatal bleeding | 15 (0.5%) | 4 (0.6%) | 1 (0.1%) | 7 (0.9%) | 3 (0.4%) | 0.615 |
|  |  |  |  |  |  |  |
| **DVT patients, Day 0 to 10** | | | | | | |
| ***Patients, N*** | ***2,558*** | ***662*** | ***671*** | ***748*** | ***477*** |  |
| Recurrent VTE | 9 (0.4%) | 5 (0.8%) | 2 (0.3%) | 2 (0.3%) | 0 | 0.061 |
| Major bleeding | 51 (2.0%) | 18 (2.7%) | 13 (1.9%) | 11 (1.5%) | 9 (1.9%) | 0.109 |
| Death | 148 (5.8%) | 41 (6.2%) | 37 (5.5%) | 42 (5.6%) | 28 (5.9%) | 0.834 |
| Fatal PE | 3 (0.1%) | 1 (0.2%) | 1 (0.1%) | 0 | 1 (0.2%) | 0.826 |
| Fatal bleeding | 9 (0.4%) | 6 (0.9%) | 1 (0.1%) | 0 | 2 (0.4%) | 0.069 |
|  |  |  |  |  |  |  |
| **DVT patients, Days 11 to 90** | | | | | | |
| ***Patients, N*** | ***2,410*** | ***621*** | ***634*** | ***706*** | ***449*** |  |
| Recurrent VTE | 34 (1.4%) | 8 (1.3%) | 11 (1.7%) | 5 (0.7%) | 10 (2.2%) | 0.714 |
| Major bleeding | 81 (3.4%) | 29 (4.7%) | 18 (2.9%) | 16 (2.3%) | 18 (4.1%) | 0.220 |
| Death | 360 (15%) | 101 (16%) | 103 (16%) | 110 (16%) | 46 (10%) | 0.011 |
| Fatal PE | 3 (0.1%) | 2 (0.3%) | 1 (0.2%) | 0 | 0 | 0.161 |
| Fatal bleeding | 20 (0.8%) | 9 (1.4%) | 5 (0.8%) | 4 (0.6%) | 2 (0.4%) | 0.085 |
|  |  |  |  |  |  |  |

***Abbreviations:*** VTE, venous thromboembolism; PE, pulmonary embolism; DVT, deep vein thrombosis.

**Supplementary Table 4. Bleeding subtypes.**

|  | **All patients** | **2005-2009** | **2010-2014** | **2015-2019** | **2020-2024** | **p for**  **trend** |
| --- | --- | --- | --- | --- | --- | --- |
| **Overall (0-90 days)** | | | | | | |
| ***Patients, N*** | ***5,763*** | ***1,437*** | ***1,499*** | ***1,559*** | ***1,268*** |  |
| Major bleeding | 293 (5.1%) | 78 (5.4%) | 73 (4.9%) | 75 (4.8%) | 67 (5.3%) | 0.630 |
| Gastrointestinal | 107 (1.9%) | 25 (1.7%) | 33 (2.2%) | 26 (1.7%) | 23 (1.8%) | 0.712 |
| Hematoma | 74 (1.3%) | 25 (1.7%) | 14 (0.9%) | 18 (1.2%) | 17 (1.3%) | 0.276 |
| Retroperitoneal | 26 (0.5%) | 7 (0.5%) | 5 (0.3%) | 8 (0.5%) | 6 (0.5%) | 0.515 |
| Intracranial | 24 (0.4%) | 5 (0.3%) | 9 (0.6%) | 6 (0.4%) | 4 (0.3%) | 0.623 |
| Other sites | 62 (1.1%) | 16 (1.1%) | 12 (0.8%) | 17 (1.1%) | 17 (1.3%) | 0.595 |
| Fatal bleeding | 54 (0.9%) | 22 (1.5%) | 9 (0.6%) | 12 (0.8%) | 11 (0.9%) | 0.172 |
| Gastrointestinal | 24 (0.4%) | 12 (0.8%) | 4 (0.3%) | 5 (0.3%) | 3 (0.2%) | 0.020 |
| Intracranial | 8 (0.1%) | 1 (0.1%) | 4 (0.3%) | 1 (0.1%) | 2 (0.2%) | 0.611 |
|  |  |  |  |  |  |  |
|  | | | | | | |
| **Days 0 to 10** | | | | | | |
| Major bleeding | 128 (2.2%) | 35 (2.4%) | 31 (2.1%) | 32 (2.1%) | 30 (2.4%) | 0.705 |
| Gastrointestinal | 52 (0.9%) | 17 (1.2%) | 16 (1.1%) | 11 (0.7%) | 8 (0.6%) | 0.076 |
| Hematoma | 33 (0.6%) | 10 (0.7%) | 6 (0.4%) | 9 (0.6%) | 8 (0.6%) | 0.860 |
| Retroperitoneal | 6 (0.1%) | 1 (0.1%) | 2 (0.1%) | 1 (0.1%) | 2 (0.2%) | 0.486 |
| Intracranial | 7 (0.1%) | 1 (0.1%) | 1 (0.1%) | 1 (0.1%) | 4 (0.3%) | 0.124 |
| Other sites | 30 (0.5%) | 6 (0.4%) | 6 (0.4%) | 10 (0.6%) | 8 (0.6%) | 0.502 |
| Fatal bleeding | 19 (0.3%) | 9 (0.6%) | 3 (0.2%) | 1 (0.1%) | 6 (0.5%) | 0.319 |
| Gastrointestinal | 10 (0.2%) | 6 (0.4%) | 3 (0.2%) | 0 | 1 (0.1%) | 0.022 |
| Intracranial | 2 (0.03%) | 0 | 0 | 0 | 2 (0.2%) | 0.132 |
|  |  |  |  |  |  |  |
| **Days 11 to 90** | | | | | | |
| ***Patients, N*** | ***5,329*** | ***1,307*** | ***1,393*** | ***1,459*** | ***1,170*** |  |
| Major bleeding | 165 (3.1%) | 43 (3.3%) | 42 (3.0%) | 43 (2.9%) | 37 (3.2%) | 0.688 |
| Gastrointestinal | 55 (1.1%) | 8 (0.6%) | 17 (1.2%) | 15 (1.0%) | 15 (1.3%) | 0.236 |
| Hematoma | 41 (0.8%) | 15 (1.2%) | 8 (0.6%) | 9 (0.6%) | 9 (0.8%) | 0.180 |
| Retroperitoneal | 20 (0.4%) | 6 (0.5%) | 3 (0.2%) | 7 (0.5%) | 4 (0.4%) | 0.738 |
| Intracranial | 17 (0.3%) | 4 (0.3%) | 8 (0.6%) | 5 (0.4%) | 0 | 0.106 |
| Other sites | 32 (0.6%) | 10 (0.8%) | 6 (0.4%) | 7 (0.5%) | 9 (0.8%) | 0.965 |
| Fatal bleeding | 35 (0.7%) | 13 (1.0%) | 6 (0.4%) | 11 (0.8%) | 5 (0.4%) | 0.314 |
| Gastrointestinal | 14 (0.3%) | 6 (0.5%) | 1 (0.1%) | 5 (0.3%) | 2 (0.2%) | 0.281 |
| Intracranial | 6 (0.1%) | 1 (0.1%) | 4 (0.3%) | 1 (0.1%) | 0 | 0.562 |
|  |  |  |  |  |  |  |

**Supplementary Figure 1. Temporal Trends in Anticoagulant Use.** **(Line or stacked area chart)**

**Early phase**

 **CrCl 15-30 mL/min	CrCl <15 mL/min or hemodialysis**

**Late phase**

 **CrCl 15-30 mL/min	CrCl <15 mL/min or hemodialysis**

**Abbreviations:** CrCl, creatinine clearance; LMWH, low-molecular-weight heparin; DOACs, direct oral anticoagulants.

**Principal Investigator**: Manuel Monreal.

**RIETE Steering Committee Members**: Paolo Prandoni, Benjamin Brenner and Dominique Farge-Bancel.

**RIETE National Coordinators**:  Raquel Barba (Spain), Peter Verhamme (Belgium), Hugo Hyung Bok Yoo (Brazil), Radovan Malý (Czech Republic), Laurent Bertoletti (France), Sebastian Schellong (Germany), Inna Tzoran (Israel), Pierpaolo Di Micco (Italy), Abilio Reis (Portugal), Marijan Bosevski (R. North Macedonia), Lucia Mazzolai (Switzerland), Joseph A. Caprini (USA), My Hanh Bui (Vietnam).

**RIETE Registry Coordinating Center**: S & H Medical Science Service.

**APPENDIX**

**Members of the RIETE Group**

**SPAIN**: Abad-Fernández A, Adarraga MD, Aibar J, Alberich-Conesa A, Alda A, Alfonso J, Alonso-Beato R, Álvarez-Albarrán J, Amado C, Angelina-García M, Arcelus JI, Ballaz A, Barba R, Barbagelata C, Barrón M, Barrón-Andrés B, Beddar-Chaib F, Blanco-Molina Á, Calvo-Pascual S, Chamorro N, Chasco L, Claver G, Crecente-Otero P, Criado J, De Juana-Izquierdo C, Del Molino F, Del Toro J, Delgado-Casado N, Demelo-Rodríguez P, Díaz-Pedroche MC, Díaz-Peromingo JA, Dubois-Silva Á, Durán D, Escribano JC, Fernández-Martínez de Septién C, Fernández-Morales M, Ferreiro-Celeiro J, Fidalgo Á, Formica A, Francisco I, Fuentes-Spínola MF, Gabara C, García-Ortega A, García-Bragado F, Gavín-Sebastián O, Gil-Díaz A, Girona E, Gómez-Cuervo C, González-Munera A, Gorostidi-Pérez J, Guirado L, Gutiérrez-Guisado J, Hernández-Blasco L, Hernández-Borge J, Jiménez D, Jou I, Joya MD, Llamas P, López-Brull H, López-Jiménez L, López-Miguel P, López-Núñez JJ, López-Rubio M, López-Ruiz A, López-Sáez JB, Lorenzo A, Madridano O, Maestre A, Marchena PJ, Martín-del Pozo M, Mas-Maresma L, Moisés-Lafuente J, Monreal M, Monzón-Escribano L, Moreno-Casas S, Moreno-Fernández A, Nieto JA, Núñez-Fernández MJ, Olid M, Ortiz M, Osorio J, Osoro-Suárez C, Otálora S, Padín-Paz EM, Pagán J, Parra-Caballero P, Parra P, Payró-Bisart N, Pedrajas JM, Pérez-Pinar M, Pérez-Ductor C, Peris ML, Pesce ML, Planes-Diez M, Prieto-Gañán LM, Puche G, Raposo S, Rivas A, Rivera-Cívico F, Rodríguez-Cobo A, Ruiz-Artacho P, Ruiz-Giménez N, Ruiz-Torregrosa P, Sánchez-Camacho M, Sancho T, Sendín V, Sidawi-Urbano T, Sierra-Palomares G, Sigüenza P, Sindín-Martín  L, Solé A, Suárez-Fernández S, Suárez-Simón A, Tascón-Rodríguez R, Toda MR, Tolosa C, Torrecillas A, Trujillo-Santos J, Tudela L, Valle R, Varona JF, Vega-Romero E, Vidal G, Villares P, You JS, **AUSTRIA**: Ay C, Nopp S, Pabinger I, **BELGIUM**: Erard M, Van Thillo

Q, Verhamme P, **BRAZIL**: Rocha AT, Yoo HHB, **COLOMBIA:** Carbonell SE, Gómez-Mesa JE, Montenegro AC, Roa J, **CZECH REPUBLIC**: Grenar P, Hirmerova J, Malý R, **FRANCE**: Accassat S, Benarroch S, Bertoletti L, Bura-Riviere A, Catella J, Chopard R, Couturaud F, Crichi B, Duong R, Espitia O, Le Mao R, Leclercq B, Mahé I, Millet G, Moustafa F, Poenou G, Sarlon-Bartoli G, **GERMANY**: Schellong S, **IRAN**: Jenab Y, Khodayari A, Sadeghipour P, Yadangi S, **ISRAEL**: Brenner B, Dally N, Tzoran I, **ITALY**: Barillari G, Basaglia M, Bilora F, Bissacco D, Bortoluzzi C, Bortoluzzi M, Brandolin B, Buso G, Casana R, Ciammaichella MM, Di Micco P, Guida A, Imbalzano E, Lambertenghi-Deliliers D, Licci A, Mastroiacovo D, Mazzarelli U, Pesavento R, Pizzuti V, Poz A, Prandoni P, Provisione M, Scarinzi P, Simeon V, Siniscalchi C, Visonà A, Vo Hong N, Zalunardo B, **LATVIA**: Bode M, Skride A, Zicans M, **MEXICO**: Flores-Ramírez C, Mendoza Romo-Ramírez MÁ, Tarín-Recéndez D, **PORTUGAL**: Fonseca S, Martins MD, Pereira-Fontes C, Soeiro B, **REPUBLIC OF NORTH MACEDONIA**: Bosevski M, Geceska M, Zdraveska M, **SWITZERLAND**: Barco S, Mazzolai L, **USA:** Angiolillo DJ, Bikdeli B, Caprini JA, Ortega-Paz L, Tafur AJ, **VIETNAM**: Bui MH, Nguyen ST, Pham KQ, Tran GB.

**ACKNOWLEDGEMENTS**

We express our gratitude to ROVI for supporting this Registry with an unrestricted educational grant. We also thank the RIETE Registry Coordinating Center, S&H Medical Science Service, for their quality control data, logistic and administrative support and Prof. Salvador Ortiz, Universidad Autónoma de Madrid, Statistical Advisor in S&H Medical Science Service, and Irene González Recio, Statistician in S&H Medical Science Service, for the statistical analysis of the data presented in this paper.
